# Capture of endogenous lipids in peptidiscs and effect on protein stability and activity

**DOI:** 10.1016/j.isci.2024.109382

**Published:** 2024-03-01

**Authors:** Rupinder Singh Jandu, Huaxu Yu, Zhiyu Zhao, Hai Tuong Le, Sehyeon Kim, Tao Huan, Franck Duong van Hoa

**Affiliations:** 1 Department of Biochemistry and Molecular Biology, Faculty of Medicine, Life Sciences Institute, University of British Columbia, Vancouver, BC V6T 1Z3, Canada; 2 Department of Chemistry, Faculty of Science, University of British Columbia, Vancouver, BC V6T 1Z3, Canada

**Keywords:** Membrane architecture, Structural biology, Protein structure aspects

## Abstract

Compared to protein–protein and protein–nucleic acid interactions, our knowledge of protein-lipid interactions remains limited. This is primarily due to the inherent insolubility of membrane proteins (MPs) in aqueous solution. The traditional use of detergents to overcome the solubility barrier destabilizes MPs and strips away certain lipids that are increasingly recognized as crucial for protein function. Recently, membrane mimetics have been developed to circumvent the limitations. In this study, using the peptidisc, we find that MPs in different lipid states can be isolated based on protein purification and reconstitution methods, leading to observable effects on MP activity and stability. Peptidisc also enables re-incorporating specific lipids to fine-tune the protein microenvironment and assess the impact on downstream protein associations. This study offers a first look at the illusive protein-lipid interaction specificity, laying the path for a systematic evaluation of lipid identity and contributions to membrane protein function.

## Highlights

• Endogenous (annular) lipids impact membrane protein stability and enzymatic activity
• Peptidisc captures membrane proteins in their intermediate lipidic states
• Exogenous and non-bilayer lipids can be added to change protein stability and activity

## Graphical abstract

**Figure:**
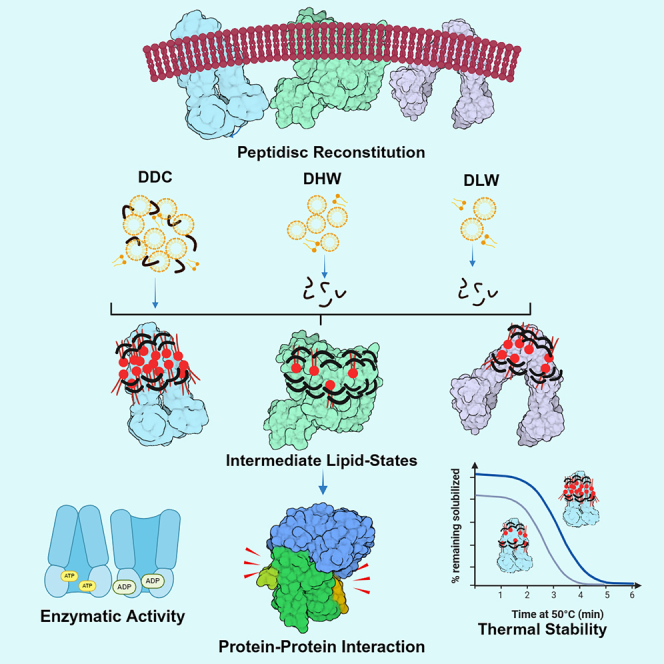

## Introduction

Incorporated in the lipid bilayer, membrane proteins (MPs) are critical for the transfer of material and information between the cytosol and the environment. In humans, dysfunction of MPs is linked to chronic diseases such as cystic fibrosis, Parkinson’s, and Alzheimer’s,^1^^,^^2^^,^^3^^,^^4^ while in bacteria, MPs are recognized as prime targets to combat rising antimicrobial resistance.^5^^,^^6^ Despite the obvious medical importance, a knowledge gap persists regarding the direct and indirect regulation of MPs by their local lipid environment.^7^ These MPs-lipids interactions are difficult to study partly due to the innate insolubility of MPs in aqueous solution, which is a rigid prerequisite for modern biochemical investigations.^8^^,^^9^

Classically, MPs have been made water-soluble by extraction from their native membrane using detergents. Detergents provide a simple and universal way to solubilize MPs, but they also strip away certain lipids that are crucial for fundamental aspects of protein biogenesis and function.^10^^,^^11^^,^^12^^,^^13^^,^^14^ Several studies show that these membrane lipids, termed endogenous or sometimes annular, associate specifically with MPs to exert effects on their structure, stability or activity.^15^^,^^16^^,^^17^ However, the exact role of these allosteric modulators remains difficult to assess because their associations with MPs are highly dynamic, exchange rapidly and are not always resolved by current structural biology techniques.^18^ Still, a more precise definition of the relationship between MPs and their associated endogenous lipids is important to better understand the organization of cell membranes and how to manipulate MPs for therapeutic benefits.

In recent years, efforts have been directed toward developing ‘membrane mimetics’ that can stabilize MPs in a water-soluble state.^19^^,^^20^^,^^21^^,^^22^^,^^23^ It is unclear however how these environments recapitulate protein-lipid associations. The nanodisc, the most commonly employed system, requires the protein of interest to be purified in detergent before a large amount of exogenous lipids is added to recreate a membrane bilayer, confounding the identification of the endogenous ones.^24^ The styrene-maleic acid (SMA) polymer allows direct MP extraction without detergent but it is unclear if only annular lipids are isolated along proteins due to the size heterogeneity and chemical variability of these surfactants.^25^^,^^26^^,^^27^ Thus, methods to capture lipids-MP associations and to measure their specific effect on MP activity merit further development.

In this study, we test if the peptidisc can capture MPs together with their endogenous lipids. The peptidisc is formed when multiple copies of a 4.5 kDa amphipathic peptide bind to the hydrophobic transmembrane surface of their target MP.^28^^,^^29^ Compared to the nanodisc, this mimetic does not require the addition of exogenous lipids during reconstitution, therefore the identification of endogenous lipids may be feasible. Additionally, it is possible to reconstitute MPs directly from the detergent extract, before protein purification.^30^^,^^31^ This method, hereafter termed detergent direct capture (DDC), simply requires the addition of an excess of peptidisc peptides to promote detergent removal, and thereby this procedure is likely to minimize the lipid-stripping effect caused by prolonged detergent incubation. Finally, structural analyses show that the peptide scaffold associates directly with the MPs transmembrane segments,^32^^,^^33^ yet the introduction of interfacial lipid during reconstitution may be feasible.

Here we use the well-characterized SecYEG translocon and the ABC transporter MsbA to show that peptidisc enables the isolation of endogenous lipids, as well as the incorporation of exogenous ones, with a remarkable impact on protein stability and ATPase activity. We envision our findings to aid researchers in generating peptidisc particles that better reflect the native lipid environment of their MPs of interest.

## Results

### Capture of the MsbA endogenous lipids

**Figure 1:**
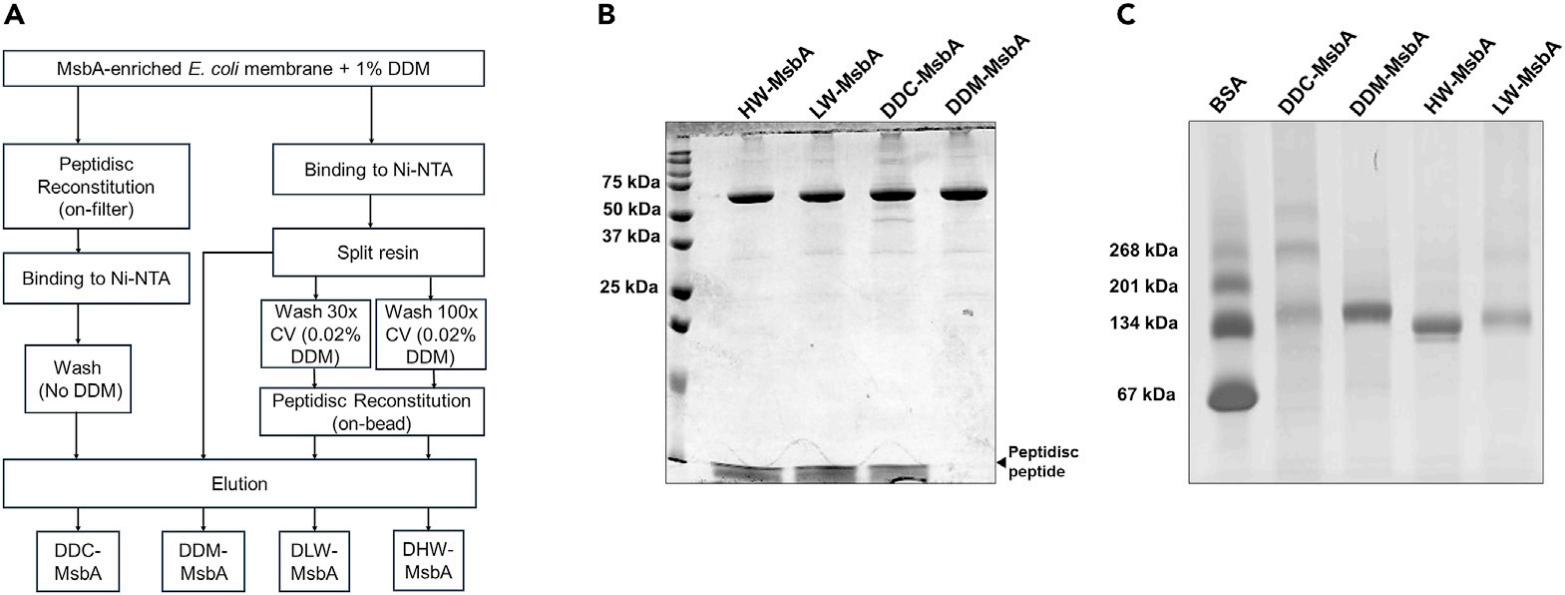
Capture of the MsbA lipidic state (A) General workflow for purifying MsbA in detergent (DDM-MsbA) or reconstituted peptidiscs via detergent direct capture (DDC-MsbA), detergent low wash (DLW-MsbA), and detergent high wash (DHW-MsbA) methods. The volume of the washing step is indicated in column volume (CV). (B) 15% SDS-PAGE analysis of the MsbA preparations followed by Coomassie blue staining of the gel. (C) 5–13% clear-native-PAGE analysis of the MsbA preparations followed by Coomassie blue staining. A reference protein ladder is loaded on the left lane on each gel.

We isolated the lipids bound to MsbA using three different methods, detergent direct-capture DDC, detergent low-wash (DLW), and detergent high-wash (DHW) (Figure 1A). In all methods, the membrane fraction bearing His_6_-tagged MsbA was first solubilized with the non-ionic detergent DDM, and the detergent-resistant aggregates were removed by ultra-centrifugation. The membrane detergent extract was then processed in three different ways. In the DDC method, MsbA was reconstituted in peptidisc by detergent dilution and ultra-filtration, followed by purification via IMAC chromatography (DDC-MsbA). In the DLW method, MsbA was purified via standard IMAC chromatography before reconstitution in peptidisc (DLW-MsbA). In the DHW method, MsbA was purified via IMAC chromatography but washed extensively with the detergent buffer (∼100 column volumes) before reconstitution (DHW-MsbA). As a control, MsbA was purified and eluted in the detergent buffer only (DDM-MsbA). Each preparation was analyzed by SDS-PAGE (Figure 1B). Visual inspection of the gel confirms that MsbA is isolated with comparable purity and yield across the preparations. Inspection of the bottom part of the gel also shows that the number of peptidisc peptides bound to MsbA is relatively similar.

The same peptidisc-MsbA preparations were also analyzed by blue native gel electrophoresis (Figure 1C). On this type of gel, the peptidisc-MsbA particles migrate at slightly different positions depending on the method, probably due to dissimilarity in their lipid content. Overall, the protein migrations are consistent with the homo-dimeric structure of MsbA. We note however that additional bands are detected in the case of DDC-MsbA preparation. Given the high-purity of the preparation (Figure 1B), these bands correspond to higher-order MsbA oligomers.

### Analysis of the Peptidisc-MsbA lipid content

**Figure 2:**
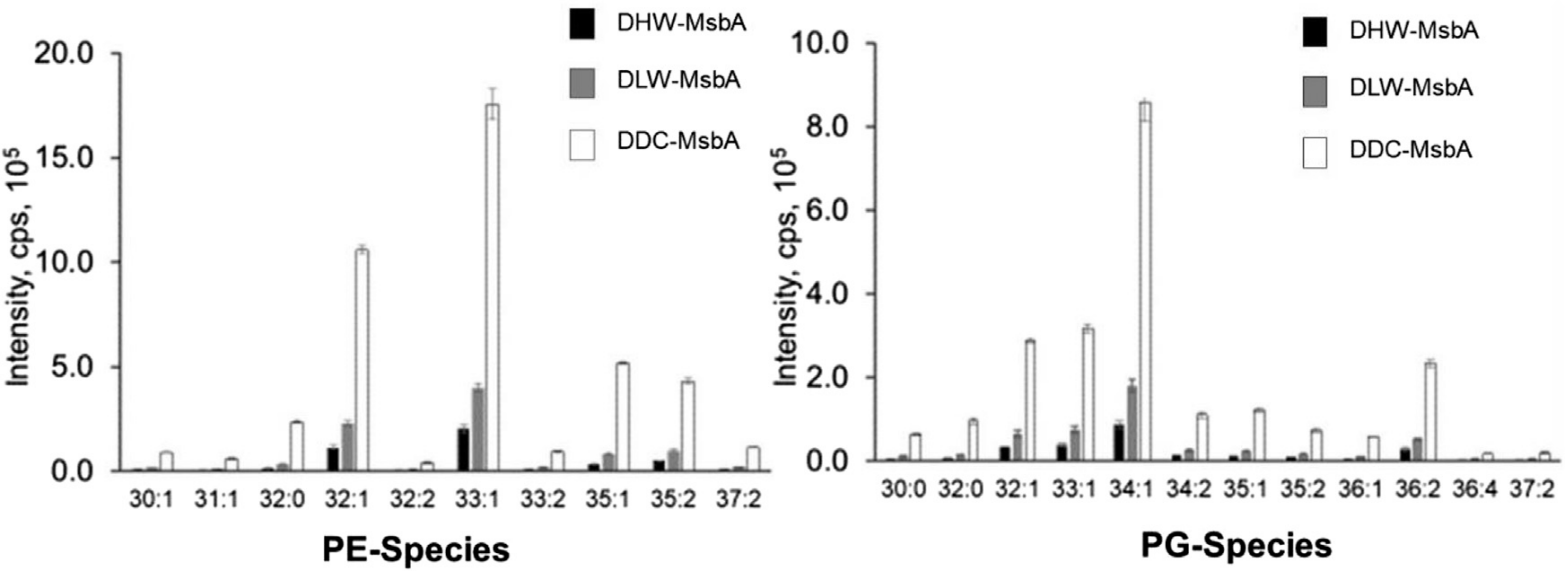
Lipid composition of the Peptidisc-MsbA preparations Relative abundance of phosphatidylethanolamine (PE) and phosphatidylglycerol (PG) species as determined by liquid chromatography and tandem mass spectrometry. Values are presented as mean ± SD (n = 3). CPS refers to the count per second.

We used reverse-phase LC-MS/MS to estimate the relative abundance of the lipids captured in the peptidisc-MsbA preparations (Figure 2). This initial lipidomic analysis was focused on the two most abundant *E. coli* lipids; the zwitterionic phosphatidylethanolamine (PE) and the anionic phosphatidylglycerol (PG), which make up 75% and 20% of the bacterial inner membrane, respectively. A representative total ion chromatogram (TIC) is presented to illustrate the effective separation of these lipids and the variety of molecular species detected in each region of the chromatogram (Figure S1). The identity of the phospholipids was then assigned using their nominal mass-to-charge ratio (*m/z*; Figure 2). The results from this analysis reveal that the relative abundance of the PE and PG lipids captured with MsbA are strikingly different across the preparations with the overall intensities decreasing from DDC-MsbA to DLW-MsbA to DHW-MsbA. The different classes of lipids are however similarly present in all three preparations: among the 10 different PE lipids, the most abundant species is the monounsaturated 32:1, and among the 12 different PG lipids, the most abundant species is the monounsaturated 34:1 (Figure 2).

### Effect of lipids on Peptidisc-MsbA ATPase activity

**Figure 3:**
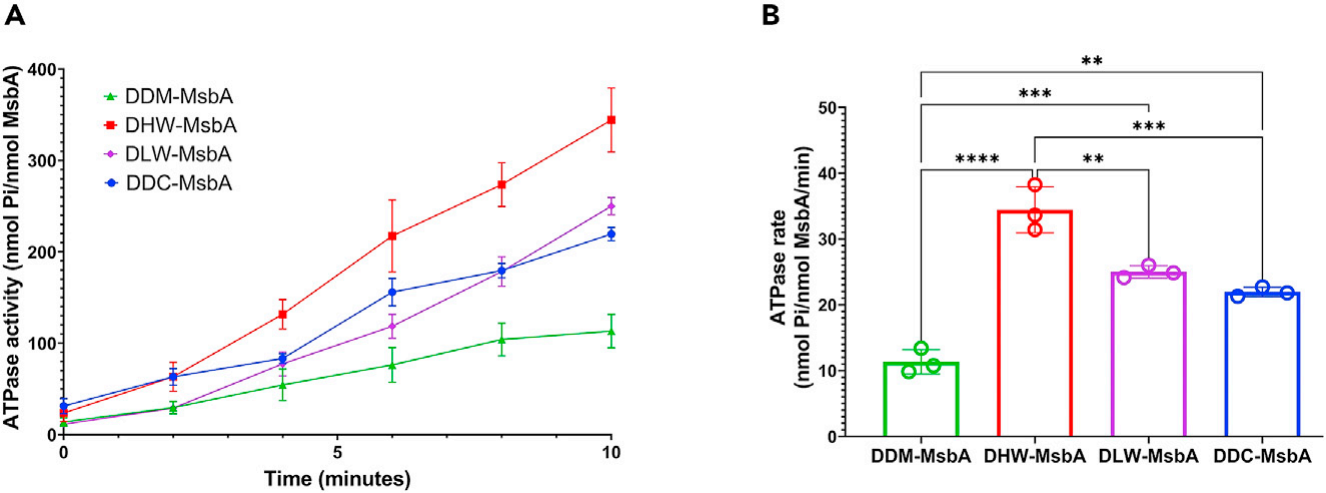
ATPase activity of the DDM-MsbA and Peptidisc-MsbA preparations (A) The ATPase activity of 5μg of peptidisc reconstituted and DDM solubilized MsbA measured in a Malachite green assay as described in the method details. (B) The rate of ATP hydrolysis was quantified for each MsbA sample over a 10 min period. The measured values are presented as mean ± SD (n = 3). Statistical analysis performed by one-way ANOVA with Tukey’s multiple comparison test comparing each group, ∗∗∗∗p < 0.0001, ∗∗∗p < 0.001, ∗∗p < 0.01.

We determined the ATPase activity of the Peptidisc-MsbA preparations, using the DDM-MsbA preparation as a reference. The measurement was performed at 37°C over a period of 10 min (Figure 3A) to estimate the initial rate of ATP hydrolysis (Figure 3B). In agreement with earlier reports, the ATPase activity of DDM-MsbA is ∼160 nmol/mg/min.^32^ The ATPase activity of the Peptidisc-MsbA is however 2- to 5-fold higher than in detergent, with the activity increasing from DDC-MsbA to DLW-MsbA to DHW-MsbA. Thus, it appears that the delipidation of MsbA prior to peptidisc reconstitution is correlated with an overall increase in its endogenous ATPase activity.

### Effect of lipids on the Peptidisc-MsbA stability

**Figure 4:**
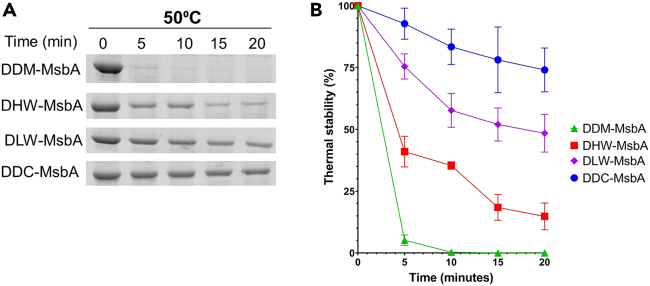
Thermal stability of the DDM-MsbA and Peptidisc-MsbA preparations (A) The preparations (20 μg each) were incubated at 50°C for the indicated time followed by ultracentrifugation at 180000*g* along with the room temperature control sample to isolate the thermostable fraction. An aliquot (15 μL) was analyzed by 15% SDS-PAGE and Coomassie blue staining. The SDS-PAGE is shown as a representative assay. (B) The thermal stability assay was performed in triplicate. The intensity of the MsbA protein bands seen on the SDS-PAGE gels were quantified using the ImageJ software and plotted as a linear time course. All values are presented as mean ± SD (n = 3).

We determined the stability of the Peptidisc-MsbA preparations using thermal denaturation. Protein aliquots were incubated at 50°C, followed by ultra-centrifugation and SDS-PAGE analysis to estimate the percentage remaining in the soluble fraction. A representative SDS-PAGE dataset, and corresponding triplicate analysis, are presented in Figures 4A and 4B, respectively. The results reveal the low thermal stability of the DDM-MsbA sample, which is consistent with its low ATPase activity (Figure 3B). Comparatively, the thermal stability of the Peptidisc-MsbA preparations is much higher (40%–80% higher than detergent), with the stability increasing from DHW-MsbA to DLW-MsbA to DDC-MsbA. Thus, it appears that the higher amount of lipids in the peptidisc preparation confers to the protein a higher thermal stability.

### Lipids bound to the SecYEG complex

**Figure 5:**
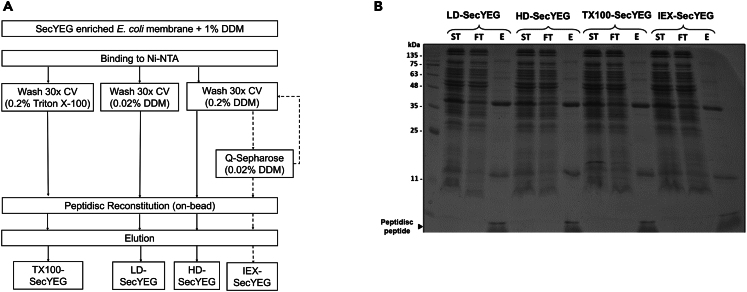
Capture of the SecYEG complex lipid state (A) General workflow for purifying SecYEG in peptidiscs after washing the complex in different detergent conditions. The washing volume is indicated in column volume (CV). (B) The purification and reconstitution efficiency of SecYEG was monitored by 15% SDS-PAGE and Coomassie blue staining. The starting material (ST), IMAC flowthrough (FT), and final elution (E) for each sample is shown.

We determined the importance of lipids on the activity of the SecYEG translocon complex. It has been reported that the functional association of this heterotrimer with the SecA ATPase depends on acid lipids, in particular cardiolipin.^34^ After membrane solubilization, the SecYEG complex was immobilized on Ni-NTA agarose and washed with 0.02% DDM (LD-SecYEG), 0.2% DDM (HD-SecYEG), or 0.2% Triton X-100 (TX100-SecYEG). To de-lipidate the complex further, the 0.2% DDM preparation (HD-SecYEG) was subject to an ion-exchange chromatographic step (IEX-SecYEG) (Figure 5A). These protein preparations were then reconstituted in peptidiscs and analyzed by SDS-PAGE. The overall SecYEG purity and yield were comparable across the different preparations (Figure 5).

**Figure 6:**
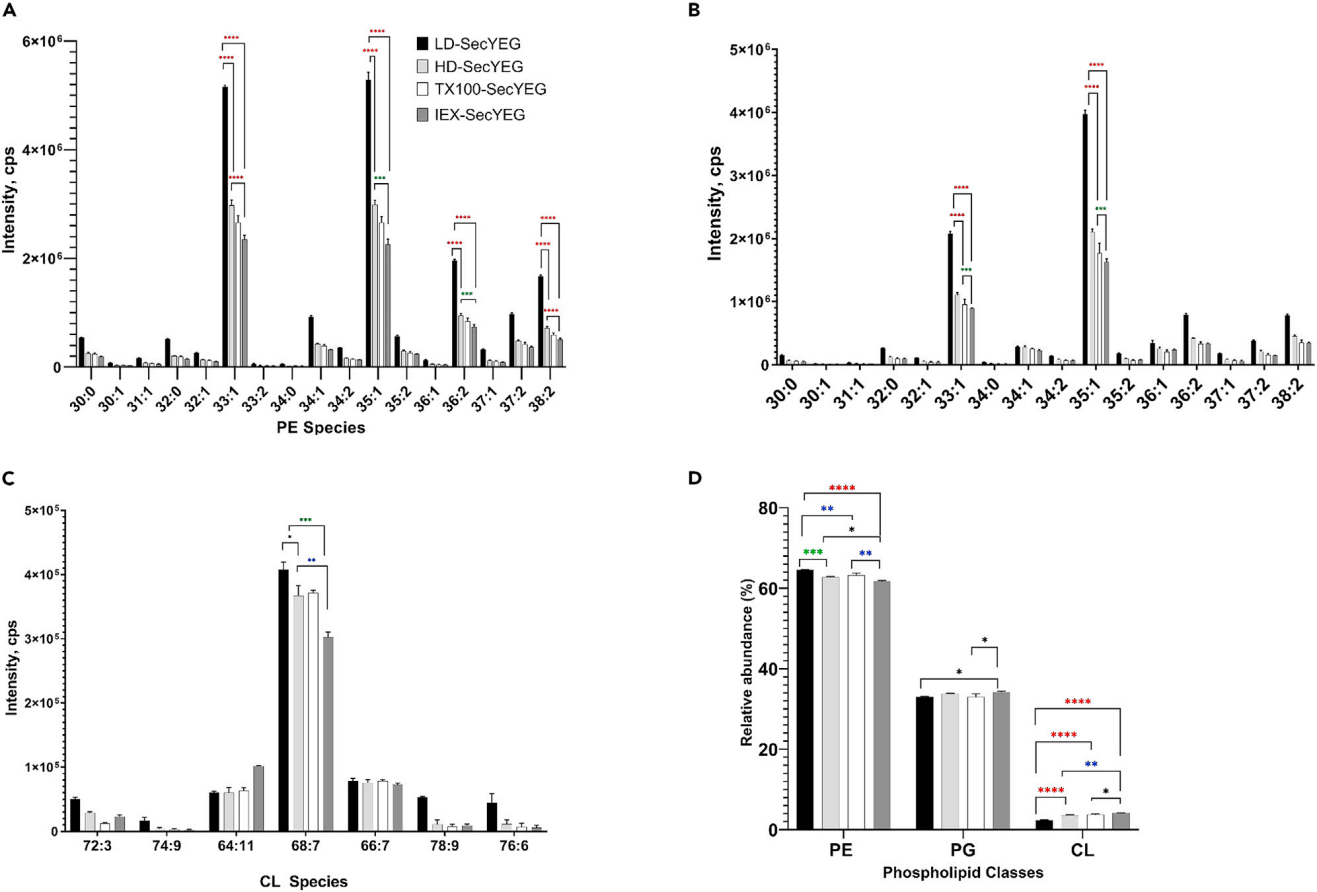
Comparison of the lipid compositions of the Peptidisc-SecYEG preparations (A–C) The relative abundance for each identified lipid species of (A) PE, (B) PG, and (C) CL are shown. (D) Relative abundance of each phospholipid class. To obtain the relative abundance in a lipid class, the summed lipid intensities for a lipid class in a Peptidisc-SecYEG reconstitution was divided by the sum of all intensities of that lipid class across all Peptidisc-SecYEG reconstitutions. The data points are presented as mean ± SD (n = 3). Statistical analysis was performed by one-way ANOVA with Tukey’s multiple comparison tests comparing each group, ∗∗∗∗p < 0.0001, ∗∗∗p < 0.001, ∗∗p < 0.01, ∗p < 0.05.

A triplicate LC-MS/MS analysis was performed in the positive mode as above to identify the PE (n = 20) and PG (n = 16) species captured in the different preparations (Figure 6). We also performed the MS analysis in reverse-phase mode to identify CL (n = 7). Interestingly, comparing the least delipidated sample (LD-SecYEG) to the most delipidated one (IEX-SecYEG), there is an evident depletion of PE and PG lipids (∼2-fold), but in comparison, the CL lipid is depleted to a much lower extent (∼0.2-fold depletion). This result suggests that CL is tightly bound to SecYEG, and this observation seems consistent with previous reports showing the importance of CL to the translocon function.^34^ We also note that the most abundant lipids bound to SecYEG (i.e., PE 33:1/35:1 and PG 33:1/35:1) were slightly different from those bound to MsbA (i.e., PE 32:1/33:1 and PG 34:1). Whether this difference in the lipid chain length impacts the structure and function of the transporters remains to be determined.

**Figure 7:**
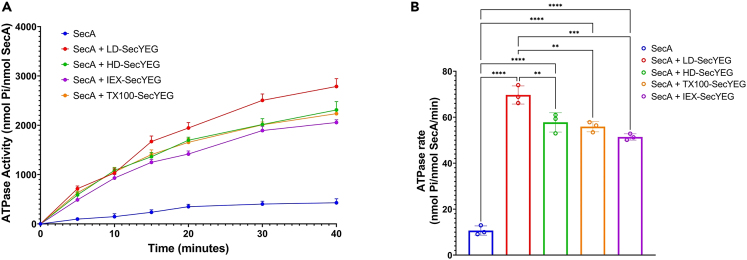
Activation of the SecA ATPase with the Peptidisc-SecYEG preparations (A) The SecA ATPase activity was measured in a Malachite green assay over a 40-min period as described in the method details. (B) The rate of ATP hydrolysis was determined for each sample in triplicate. All values are presented as a mean ± SD (n = 3). Statistical analysis was performed by one-way ANOVA with Tukey’s multiple comparison tests comparing each group, ∗∗∗∗p < 0.0001, ∗∗∗p < 0.001, ∗∗p < 0.01.

We then measured the effect of the SecYEG-bound lipids on the SecA binding and ATPase activities. These assays are possible since the translocon is water-soluble when stabilized peptidiscs. We performed a time-course experiment at 37°C (Figure 7) to estimate the initial rate of ATP hydrolysis. A modest (∼10–15%) reduction in ATPase activity was obtained as the SecYEG preparation is delipidated (LD-SecYEG > HD-SecYEG> IEX-SecYEG). We also used clear-native gel electrophoresis to estimate the relative binding affinity of SecA to the peptidisc-SecYEG preparations, but no significant difference in the amount of SecA bound to SecYEG was observed (Figure S2).

### Introduction of lipids in the Peptidisc-SecYEG preparation

**Figure 8:**
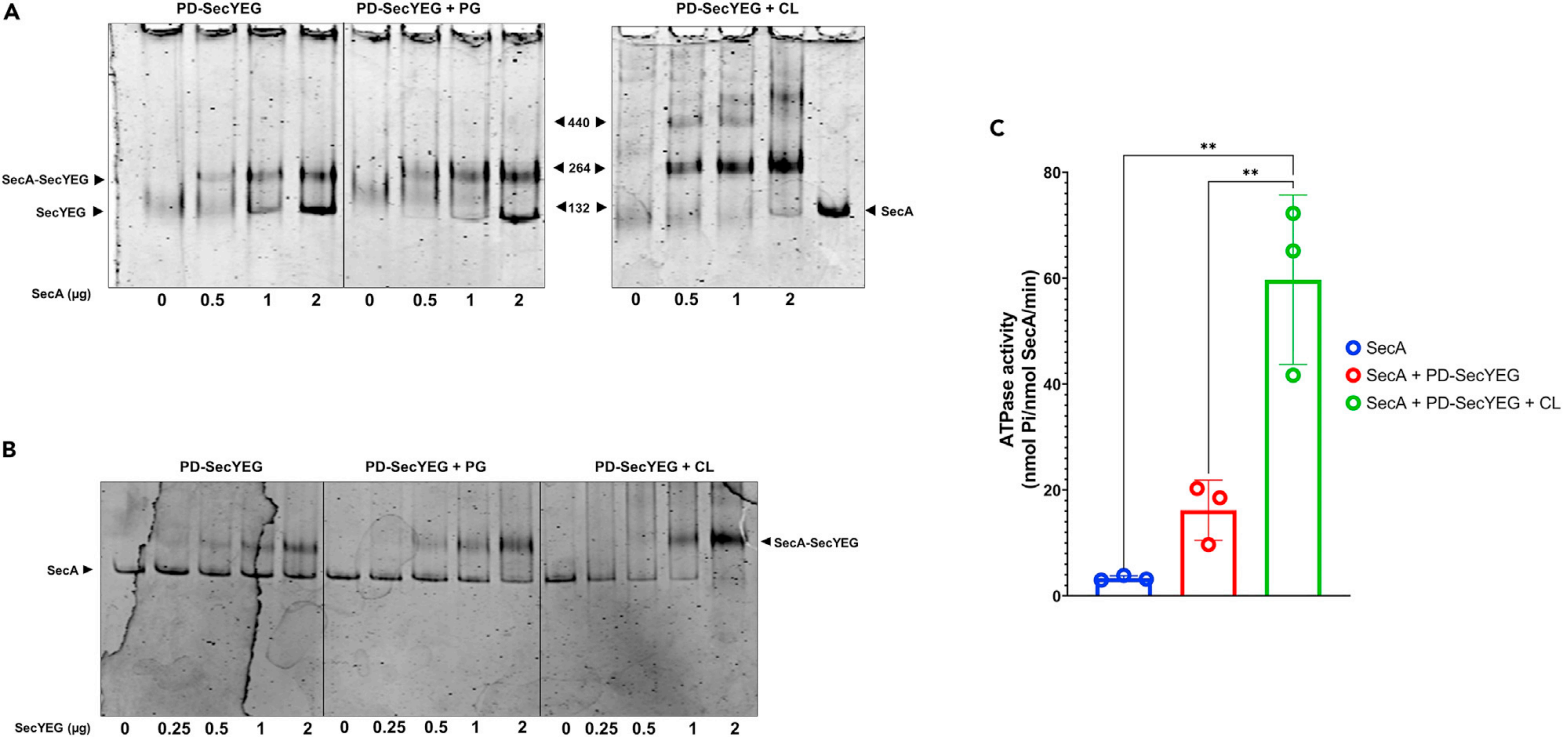
Effect of lipids on SecA-SecYEG complex formation and ATPase activity (A) The indicated Peptidisc-SecYEG preparations (1μg each) were incubated on ice with the indicated amount of SecA (0–2 μg) before detection of the complex by 5–13% clear-native PAGE and Coomassie blue staining of the gel. (B) The indicated amounts of the Peptidisc-SecYEG preparations (0–2 μg) were incubated with a constant amount of SecA (1μg) before analysis by 5–13% clear-native PAGE. (C) The SecA ATPase activity in the presence of the indicated Peptidisc-SecYEG preparations was measured in a Malachite green assay and quantified at 660nm. All values are presented as a mean ± SD (n = 3). Statistical analysis performed by one-way ANOVA with Tukey’s multiple comparison test comparing each group, ∗∗p<0.01.

The peptidisc is unable to support a lipid bilayer as the nanodisc does, yet the incorporation of exogenous lipids, which may be important to protein function, is feasible. The delipidated SecYEG preparation (HD-SecYEG) was incubated with minute amounts of PG or CL lipids (20:1 lipid to SecYEG molar ratio) before reconstitution in peptidisc. To remove unincorporated lipids, the preparations were purified by size exclusion chromatography (Figure S3). The effect of the exogenous lipids on the SecA-SecYEG binding affinity was then determined by reciprocal titration on clear-native gel electrophoresis. Evidently, the presence of additional CL lipids in the Peptidisc-SecYEG particles (labeled PD-SecYEG-CL) dramatically increases the binding affinity of SecA for SecYEG, as shown by the appearance of a SecA-SecYEG complex on the gels (Figure 8A). Some minor species are also detected near the top of the gel, which probably reflects the CL-dependent dimeric association of the SecYEG complex as previously reported.^35^^,^^36^ In agreement with the binding results above, an almost ∼ 4-fold increase in the SecA ATPase activity was obtained when the translocon is reconstituted with CL compared to without (Figure 8B). Thus, the incorporation of exogenous lipids to the peptidisc-stabilized SecYEG allows to recapitulate the CL-dependency of the SecYEG-SecA interaction.

## Discussion

During MP purification, prolonged exposure to the detergent micelles progressively strips away bound lipids, a process known as delipidation.^37^^,^^38^ We exploit this property here to isolate MPs containing various amounts of endogenous lipids, and we capture these lipid intermediate states in peptidiscs using three methods. In the Detergent Direct Capture (DDC) method, the target protein is reconstituted immediately after detergent extraction to maximize the chance of capturing its naturally bound lipids. In the Detergent Low Wash and High Wash methods (DLW and DHW), the protein is exposed to increasing amounts of detergent to maximize its delipidation before reconstitution. These workflows allow us to assess the impact of endogenous lipids on protein stability and activity.

With the ABC transporter MsbA, the DDC method allows to capture ∼10 times more PE and PG lipids than with the DHW method (Figure 2). As a direct consequence, the thermal stability of MsbA is dramatically increased, with ∼75% of the DDC-MsbA remaining soluble after a 20-min incubation at 50°C compared to only ∼15% when MsbA is delipidated (Figure 3). The lipidomic analysis further showed that the different PE and PG lipid classes were equally depleted during the detergent treatment, suggesting that MsbA does not have particular lipid specificity. We note that Lipid A was not detected in our MS analysis because identification of this glycolipid requires a specific acid hydrolysis step.^39^ We reported however that Lipid A is present at the MsbA homodimeric interface in the DHW-MsbA preparation,^32^ we thus anticipate the presence of the lipid in the other preparations as well. Further analysis will be required to determine the exact number of lipid molecules bound to MsbA in the DDC preparation but as is, the lipid-mediated increased thermal stability we report is a significant finding because it indicates that the capture of MPs directly from the initial lipid-detergent extract, before purification, is an effective way to increase protein stability and thereby the protein downstream usability.

The presence of endogenous lipids increased the thermal stability of MsbA, yet these additional lipids also lowered the transporter ATPase activity. It is well known that MsbA ATPase depends on the closure of its two nucleotide-binding domains which is coupled to the opening of its two transmembrane domains, termed the outward-facing (OF) conformation.^40^ In our previous work, we showed that delipidated MsbA in peptidisc is stabilized in this OF conformation, which is consistent with the higher ATPase activity obtained with this sample.^32^ We therefore predict here that the high lipid content of the DDC-MsbA preparation lowers the MsbA ATPase activity because lipids stabilize the transporter in the inward-facing (IF) conformation. This assumption seems supported by a recent structural analysis conducted in *E. coli* showing that MsbA exists mostly in the IF conformation when embedded in the native membrane environment.^41^ While it is unlikely that the natural lipid bilayer constraint is reconstituted in peptidisc, the current result clearly shows that the MsbA activity is directly influenced by the lipid environment. Finally, we note that MsbA is often overproduced in the membrane before purification, and the consequence of this overproduction on protein-lipid associations is unknown. Specifically, our native-gel analysis shows that protein oligomers are captured when MsbA is purified using the DDC method (Figure 1C). These oligomers were also obtained when overproduced MsbA was extracted from the membrane using the SMA polymer,^42^ indicating the tendency of this protein to cluster together in the membrane environment.

With the SecYEG translocon, we first delipidated the complex with detergent before reconstitution. This procedure allowed us to probe the translocon activity before and after the addition of exogenous lipids (Figure 5). A two- to three-fold reduction in the relative intensity of SecYEG-bound PG and PE lipids was obtained using this delipidation procedure (*i.e*., compare LD-SecYEG to IEX-SecYEG), but comparatively, the amount of SecYEG-bound CL remained relatively constant even after extensive detergent washing steps (Figure 6D). This preferential affinity of CL to the translocon has been reported before, but the conclusion was reached with indirect binding assays.^35^ Here, the controlled titration of CL during the translocon reconstitution gives direct evidence that CL increases SecA binding and activation (Figure 8). The reintroduction of a few CL molecules may allow for their remigration to sites they were displaced from during detergent solubilization. This dynamic exchange of lipids to binding pockets critical for protein function is being increasingly documented.^43^^,^^44^^,^^45^ Structural analysis will be necessary but here, the possibility to re-introduce specific lipids provides another distinctive advantage to the peptidisc membrane mimetic. For comparison, the contribution of CL would be more challenging to determine in nanodisc because this membrane mimetic requires a larger amount of lipids to create a lipid bilayer that precisely matches the protein scaffold diameter, whereas CL is a non-bilayer-forming lipid. Interestingly, it has been reported that CL promotes the dimerization of the SecYEG complex^35^^,^^36^ and these CL-dependent oligomers were also seen when CL is added to the reconstitution (Figure 8A). The capture of these larger MP oligomers is possible in peptidisc because the number of peptide scaffolds adapts spontaneously to the diameter of the target proteins.

In conclusion, we find the peptidisc a useful tool to examine the membrane lipid content and the lipid contribution to MP structure and function. We note that several recent studies have employed the peptidisc scaffold, and these peptidisc-stabilized proteins have been used for structural analysis of mammalian,^46^^,^^47^^,^^48^ viral^49^ and bacterial MPs,^32^^,^^50^^,^^51^ as well as for ligand binding,^33^^,^^52^^,^^53^^,^^54^ and protein-protein interaction studies.^30^^,^^55^^,^^56^ These structural and functional protein analyses, combined with a systematic determination of their lipids content, should help to obtain a better understanding of the allosteric effect of lipids on MPs.

### Limitations of the study

The precise quantity of lipid molecules engaged with the MPs within the DDC preparation remains undetermined, leaving a knowledge gap about how these lipids influence the structure of the peptidisc particles. Furthermore, despite lipidomic analysis revealing an enrichment of cardiolipin with the SecYEG complex, the rationale behind this preferential detection could be linked to the positioning of the complex in cardiolipin-rich regions of the bacterial envelope. Moreover, the inquiry into whether the captured lipids are genuinely “annular” or simply “endogenous” persists as an open question, not solely pertinent to this investigation but also across the broader field. These unresolved aspects underscore the need for further research to unravel the intricacies of lipid-protein interactions and their implications in cellular membranes.

## STAR★Methods

### Key resources table

**Table:** 

REAGENT or RESOURCE	SOURCE	IDENTIFIER
**Bacterial and virus strains**		

*E. coli* BL21 (DE3)	Lab stock	N/A

**Chemicals, peptides, and recombinant proteins**		

Peptidisc (NSPr)	Peptidisc Biotech	www.peptidisc.com
Dodecyl-β-d-maltoside (DDM)	Anatrace	D310
Triton X-100	Anatrace	T1001
DOPG	Avanti Polar	840475
Cardiolipin	Avanti Polar	710333; 710335
Ampicillin	Gold Bio	A-301
Kanamycin	Gold Bio	K-120
IPTG	Gold Bio	I2481
Malachite Green	Sigma	M9015
Tris	Bio Shop	TRS001
Yeast Extract	Bio Shop	YEX401
Tryptone	Bio Shop	TRP402
NaCl	Bio Shop	SOD004
Imidazole	Bio Shop	IMD508
Acrylamide (40%)	Bio Shop	ACR006
Bis-acrylamide (2%)	Bio Shop	ACR007
TEMED	Bio Shop	TEM001
Ammonium persulfate	Fisher Scientifc	BP179
ATP	Bio Shop	ATP008

**Recombinant DNA**		

pET28-his_6_MsbA	Lab stock	N/A
pET28-his_6_Syd	Lab stock	N/A
pBad22-his_6_EYG	Lab stock	N/A
pET28-SecAhis_6_	Lab stock	N/A

**Software and algorithms**		

ImageJ	ImageJ	www.imagej.net
GraphPad Prism	Prism - GraphPad	www.graphpad.com

### Resource availability

#### Lead contact

Further information and requests for resources and reagents should be directed to and will be fulfilled by the lead contact, Franck Duong (fduong@mail.ubc.ca).

#### Materials availability

This study did not generate unique reagents.

#### Data and code availability

• All data reported in this paper will be available from the lead contact upon reasonable request.
• This paper does not report the original code.
• Any additional information required to reanalyze the data reported in this paper is available from the lead contact upon request.

### Experimental model and study participant details

*E. coli* BL21 (DE3) strain was used in this study. For all related experiments, *E. coli* cells (from the glycerol stock at -80°C freezer) were used as the starting material and grown in fresh LB broth media.

### Method details

#### Plasmids and reagents

*E. coli* strain BL21 (DE3) is from our laboratory collection. Plasmids encoding His-tagged MsbA (pET28-his_6_MsbA), His-tagged SecA (pET28-SecAhis_6_), His-tagged Syd (pET28-his_6_Syd) and His-tagged SecYEG (pBad22-his_6_EYG) were constructed earlier in our laboratory.^30^^,^^57^^,^^58^^,^^59^ Peptidisc peptides (NSPr, purity >90%) were obtained from Peptidisc Biotech. Detergents *N*-dodecyl-β-d-maltoside (DDM) and Triton X-100 (TX100) were purchased from Anatrace. Lipids DOPG (18:1 (Δ9-Cis) 1,2-dioleoyl-sn-glycero-3-phospho-(1′-rac-glycerol) and CL (16:0-18:1; 1',3'-bis[1-palmitoyl-2-oleoyl-sn-glycero-3-phospho]-glycerol) were purchased from Avanti Polar Lipids. Nickel-chelating Sepharose (Ni-NTA resin) was obtained from Qiagen. Superdex 200, Tricorn columns and Q Sepharose resins were from GE Healthcare. Tryptone, yeast extract, NaCl, imidazole, Tris-base, acrylamide 40%, bis-acrylamide 2%, and TEMED were obtained from Bioshop Canada. Isopropyl β-d-1-thiogalactopyranoside (IPTG), arabinose, and kanamycin were purchased from GoldBio. For LC-MS/MS experiment, methanol (MeOH), water (H_2_O), acetonitrile (ACN), isopropyl alcohol (IPA), sodium formate (NaFA), formic acid, ammonium formate (all LC−MS-grade) and methyl tert-butyl ether (MTBE, LC grade) were purchased from Thermo Fisher Scientific (Waltham, MA, USA). Ammonium hydroxide was obtained from MilliporeSigma (Burlington, MA, USA). All other chemicals were obtained from Fisher Scientific Canada.

#### Protein expression, purification, and membrane preparation

His-tagged MsbA was produced in *E. coli* BL21(DE3) at 37°C in 1L of LB medium supplemented with 50 μg/mL kanamycin. The inducer IPTG (0.5 mM) was added during the exponential growth phase (OD_600nm_ ∼ 0.4). After 3 hours, cells were harvested by low-speed centrifugation (6000*g*, 6 min) and resuspended in Buffer A (50 mM Tris-HCl pH 7.8, 100mM NaCl, 10% Glycerol) supplemented with phenylmethylsulfonyl fluoride (1 mM). Cells were lysed through a microfluidizer (Microfluidics; 3 passes at 15,000 psi at 4°C). Unbroken cells and large aggregates were removed by low-speed centrifugation (6000*g*, 6 min). The crude membrane fraction containing MsbA was isolated by ultracentrifugation (100000*g*, 45 min, 4°C, Beckman Coulter rotor Ti70). Membranes were resuspended in Buffer A at 5 mg/mL and stored at -70°C for later use. The His-tagged SecYEG was produced in *E. coli* BL21(DE3) at 37°C in 3L of LB medium supplemented with 50 μg/mL ampicillin. Protein expression was induced with 0.2% arabinose for 3 h at 37°C at an OD_600nm_ of 0.4. Cell lysis and membrane isolation were performed as described above. As a pre-purification step, membranes were layered over a sucrose gradient (11 mL 20–50% sucrose in Buffer A) and re-isolated by ultracentrifugation (200000*g*, 120 min, 4°C, Beckman Coulter SW41 rotor). The membrane fraction was recovered as a distinct brown band located in the middle of the gradient. Sucrose was diluted with 4 volumes of Buffer A. Membranes were re-isolated by ultracentrifugation (100000*g*, 45 min, 4°C, Beckman Coulter rotor Ti70), resuspended in Buffer A at 5 mg/mL and stored at -70°C for later use. His-tagged SecA and His-tagged Syd were produced in the cytosol of *E. coli* BL21(DE3) grown at 37°C in 1L of LB medium supplemented with 50 μg/mL kanamycin. Cells were induced with IPTG (0.5 mM) and lysed as described above. Proteins were purified by Ni^2+^-chelating chromatography (Ni-NTA) followed by gel filtration on a Superdex 200 HR10/30 column equilibrated in TSG buffer (50 mM Tris pH 7.9, 50 mM NaCl, 10% glycerol and 1 mM DTT), as previously described.^59^

#### Reconstitution of MsbA in peptidiscs

In the Detergent Direct Capture (DDC) method, about 3 mg of MsbA-enriched membranes were solubilized with 1% DDM (w/v) for 30 min at 4°C. The detergent extract was clarified by ultracentrifugation (180000*g*, 15 min, 4°C) and an aliquot (1 mL) of the detergent extract (2 mg/mL) was mixed with a molar excess of peptidisc peptides (1 mg). The mixture was then rapidly diluted into 15 mL of Buffer A before concentration over a nitrocellulose filter to a final volume of 500 μL (Millipore; 100 kDa cut-off, 3000*g*, 20 min, 4°C). The dilution and concentration process were repeated three times to decrease the detergent concentration to a calculated estimate of ∼0.008% final. The mixture was then incubated on a tabletop rocker with the Ni-NTA resin (150 μL) for 1 hour at 4°C. The resin was sedimented (3000*g* for 3 min) and washed once with 10 10-column volume (CV; 1.5 mL of Buffer A). The peptidisc-reconstituted MsbA (DDC-MsbA) was eluted in 200 μL of Buffer A + 600 mM Imidazole. The protein samples were directly used for downstream thermal stability and ATPase assays or stored at -20°C before lipid analysis. In the Low-Wash and High-Wash methods, an aliquot (1 mL) of the detergent extract prepared above (2 mg/mL) was incubated with Ni-NTA resin (150 μL) for 1 hour at 4°C on a tabletop rocker. The resin was then sedimented and washed three times (DLW-MsbA) or ten times (DHW-MsbA) with 1.5 mL Buffer A supplemented with 0.02% DDM (*i.e*. 30 CV and 100 CV washes, respectively). The resin was then resuspended in 1 mL of Buffer A supplemented with the peptidisc peptides (1 mg). The resin was washed with 1.5 mL Buffer A to remove excess peptide. The peptidisc-reconstituted MsbA was eluted in Buffer A + 600 mM Imidazole (200 μL). Protein samples were directly used or placed at -20°C for short term storage. In the detergent purified method, the Low-Wash protocol was followed minus the peptidisc reconstitution step. The Ni-NTA bound MsbA is then eluted in Buffer A + 600 mM Imidazole + 0.02% DDM (w/v) to generate DDM-MsbA.

#### Reconstitution of SecYEG in peptidiscs

An aliquot (1 mL) of the SecYEG membrane preparation above (5 mg/mL) was solubilized with 1% DDM in Buffer A for 1 hour at 4°C. The insoluble material was removed by ultracentrifugation (180000*g*, 15 min, 4°C) before incubation with the Ni-NTA resin (150 μL) for 30 min on a tabletop rocker at 4°C. The resin was washed three times with 1.5 mL (*i.e.* 30 CV) of either Buffer A supplemented with 0.02% DDM (LD-SecYEG), or 0.2% DDM (HD-SecYEG), or 0.2% Triton X100 (TX100-SecYEG). The resin was then resuspended in 1 mL of Buffer A supplemented with 1 mg peptidisc peptides. After 10 min at 4°C, the resin was washed twice with 1.5 mL Buffer A. The peptidisc-reconstituted SecYEG was eluted in 200 μL of Buffer A supplemented with 300 mM Imidazole. For the IEX-SecYEG sample, the SecYEG complex was eluted from the Ni-NTA resin in 300 μL Buffer A + 0.2% DDM + 300 mM imidazole. The eluate was incubated with a Q Sepharose Fast Flow resin (50 μL) and washed four times with 1 mL of Buffer C (50 mM Tris-HCl pH 7.8, 10% glycerol, 0.02% DDM) before elution in Buffer D (50 mM Tris-HCl pH 7.8, 500 mM NaCl, 10% glycerol, 0.02% DDM). The sample was loaded back onto the Ni-NTA resin (50 μL) in Buffer A + 0.02% DDM before reconstitution in peptidiscs as described above. To verify the successful reconstitution, aliquots of each preparation (5 μg) were analyzed by SDS-PAGE, BN-PAGE and CN-PAGE as indicated. All protein samples were stored at 4°C until further analysis.

#### Phospholipids addition to SecYEG in peptidiscs

Manufacturer-weighted PG and CL were dissolved in chloroform and dried under a stream of nitrogen. The dried lipids were resuspended in Buffer A (50 mM Tris pH 7.8, 100 mM NaCl, 10% glycerol) containing 0.5% DDM to a final concentration of 5 mg/mL and stored at -80°C until use. The reconstitution experiment involved mixing together the purified HD-SecYEG complex (100 μg) with PG lipid (19.5 μg) or CL lipid (40 μg) to obtain a molar ratio protein to lipid of 1:20. After 30 min incubation on ice, the peptidisc NSPr peptide (200 μg) was added to obtain a protein to peptide mass ratio of 1:2. After 30 min incubation on ice, the mixture was applied onto a Superdex S200 Tricon 10/100 column equilibrated with Buffer A to remove protein aggregates, free peptides and unbound lipids. The fractions containing the reconstituted SecYEG complex were identified by SDS-PAGE, pooled together and stored at 4°C until further analysis.

#### Lipid extraction

For each sample, a total of 270 μL of ice-cold methanol was added followed by vortexing for 10 seconds. A 10 μL of mixed internal standards containing PE 17:0/17:0 (1.5 mg/ml), CL 14:0/14:0/14:0 (1 mg/ml) were added for quality control purposes. The sample was incubated overnight at - 20°C for complete protein precipitation. Then, the solution was mixed with 900 μL of MTBE, vortexed for 10 seconds, and shaken for 5 min for lipid extraction. Subsequently, a total of 315 μL of water was added to induce the phase separation. The sample was then centrifuged at 14,000 rpm for 15 min at 4°C. The upper lipid layer was transferred to a new 1.5 mL Eppendorf vial and dried down via SpeedVac. The dried lipids were reconstituted in 100 μL of IPA/ACN solution (50:50, vol: vol) with internal standards. The reconstituted sample was vortexed and centrifuged at 14,000 rpm for 15 min at 4°C to further remove insoluble particles before LC-MS analysis.

#### Liquid chromatography and mass spectrometry analysis

An UHR-QqTOF (Ultra-High Resolution Qq-Time-Of-Flight) Impact II (Bruker Daltonics, Bremen, Germany) mass spectrometry interfaced with an Agilent 1290 Infinity II Ultrahigh-Performance Liquid Chromatography (UHPLC) system (Agilent Technologies, Santa Clara, CA, USA) was used for lipidomics analysis. LC separation was achieved using a Waters reversed phase (RP) UPLC Acquity BEH C18 Column (1.7 μm, 1.0 mm ×100 mm, 130 Å) (Milford, MA, USA) maintained at 20°C. For ESI positive mode, the mobile phase A was 60% ACN in H_2_O and the mobile phase B was IPA: ACN = 9:1, both containing 5 mM ammonium formate (pH = 4.8, adjusted by formic acid). For ESI negative mode, the mobile phase A was 60% ACN in H_2_O and the mobile phase B was IPA: ACN = 9:1, both containing 5 mM ammonium formate (pH = 9.8, adjusted by ammonium hydroxide). The LC gradient was set as follows: 0 min, 5% B; 8 min, 40% B; 14 min, 70% B; 20 min, 95% B; 23 min, 95% B; 24 min, 5% B; 33 min, 5% B. The flow rate was 0.1 mL/min. The injection volume was optimized to 2 μL for positive and negative ion mode. The ESI source conditions were set as follows: dry gas temperature, 220°C; dry gas flow, 7 L/min; nebulizer gas pressure, 1.6 bar; capillary voltage, 4500 V for positive mode and 3000 V for negative mode. The MS1 analysis was conducted using the following parameters: mass range, 50-2500 *m/z*; spectrum type: centroid, calculated using maximum intensity; absolute intensity threshold: 250. Data-dependent acquisition (DDA) MS/MS analysis parameters: collision energy: 16-30 eV; cycle time, 3 s; spectra rate: 4 Hz when intensity < 10^4^ and 12 Hz when intensity > 10^5^, linearly increased from 10^4^ to 10^5^. External calibration was applied using sodium formate to ensure the m/z accuracy before sample analysis.

#### ATPase assays

The ATPase activities were determined using the Malachite green assay^60^ with few modifications. For peptidisc reconstituted MsbA, 5 μg was incubated at 37°C in 200 μl of reaction buffer (50 mM Tris-HCl pH 7.8, 100 mM NaCl, 5 mM MgCl_2_) containing 2 mM of ATP, plus 0.02% DDM in the case of the DDM-MsbA assay. Aliquots (20 μL) were taken every two minutes and mixed with 500 μL of Malachite green solution containing 0.05% Triton X-100. Light absorption at 660 nm was measured after 10 min incubation at room temperature. The ATPase activity was calculated using a standard curve generated with a phosphorous standard solution. For SecA, 1 μg of the protein was incubated with 4 μg of peptidisc-reconstituted SecYEG in 70 μL of Translocation Buffer (50 mM Tris-HCl, pH 8.8, 50 mM NaCl, 50 mM KCl, 10 mM MgCl_2_, 1 mM DTT) containing 1 mM ATP at 37°C. Aliquots (5 μL) were mixed with 500 μL of Malachite green containing 0.05% Triton X-100 over a time course of 40 min. Light absorption was measured at 660 nm and activity was calculated using a standard curve as above. The ATPase measurements were performed in triplicate to establish the standard deviation (SD). Initial rates were calculated using the linear part of the curve and dividing the molar amount of free phosphate produced by the molar amount of MsbA or SecA over a set period (10 min for MsbA and 20 min for SecYEG).

#### Thermal stability assays

The peptidisc reconstituted protein sample concentration was adjusted to 0.5 mg/mL in Buffer B (50mM Tris-HCl pH 7.8, 100mM NaCl). For DDM-MsbA, Buffer B contained 0.02% DDM. Aliquots (50 μL) were placed in 200 μL thin-wall PCR tubes (Diamed) and placed in a thermocycler with the block and lid temperatures set to 50°C. Samples were removed from the heat block every two minutes over a time course of 10 minutes. Samples were transferred to a 1.5 mL polypropylene tube before ultracentrifugation (180000*g*, 15 min, 4°C, Beckman Coulter rotor TL-55). The soluble fraction (15 μL) obtained for each time point was analyzed by SDS-PAGE and stained with Coomassie Blue. Densitometry of the protein band intensity was performed using the software Image J.

#### Other methods

Protein concentrations were determined using the Bradford reagent (Bio-Rad). Blue-native gels, clear-native gels and electrophoresis conditions were performed as described by Schagger et al.^61^ Briefly, equal volumes of 5% and 13% acrylamide solutions were prepared in advance. Linear gradient gels were formed by mixing the two solutions. The cross-linking agents, TEMED and ammonium persulfate, were added immediately before gradient mixing. For clear-native PAGE, anode and cathode buffers consisted of Native running buffer (37 mM Tris-HCl; 35 mM Glycine; pH 8.8). For blue-native PAGE, anode buffer consisted of Native running buffer + 180 μM Coomassie Blue G-250, and cathode buffer contained Native running buffer only.

### Quantification and statistical analysis

Raw LC-MS data were converted to Analysis Base File (ABF) format using Reifycs Abf Converter (ver. 4.0.0). The converted files were processed using MS-DIAL (ver. 4.00) for peak detection, peak alignment and lipid identification.^62^ Data processing parameters in MS-DIAL are: MS1 tolerance, 0.01 Da; MS/MS tolerance, 0.05; mass slice width, 0.05 Da; smoothing method, linear weighted moving average; smoothing level, 3 scans; minimum peak width, 5 scans. Lipid identification in MS-DIAL was performed by matching experimental precursor *m/z*, isotopic ratio, and MS/MS spectrum against libraries. Lipid identification used libraries embedded in MS-DIAL. Statistical analysis was performed in Microsoft Excel including two-sided Welch's *t*-test. To compare the proportion of lipids found in each SecYEG reconstitution, we assessed the relative abundance of each phospholipid class. To do so, the lipid intensities of a single class (PE, PG, or CL) in one SecYEG reconstitution were summed and divided by its abundance across all reconstitutions. This ratio was multiplied by 100 to obtain the proportion of PE, PG, or CL found within each SecYEG reconstitution. The equation is presented:

$$\frac{\sum \left(Lipid\;species\;intensities\;in\;a\;single\;SecYEG\;reconstitution\right)}{\sum \left(Lipid\;class\;intensity\;across\;all\;SecYEG\;reconstitutions\right)}\times 100$$

Statistical analysis found in the figure legends was carried out using GraphPad Prism software version 9.0.0.
