# Supplementary material for: Capture of endogenous lipids in peptidiscs and effect on protein stability and activity
Source: iScience. 2024 Mar 1;27(4):109382. doi: 10.1016/j.isci.2024.109382 (PMC10993126; doi:10.1016/j.isci.2024.109382)
Supplement: Document S1. Figures S1–S3 [file mmc1.pdf]

iScience, Volume *27*

Supplemental information

Capture of endogenous lipids in peptidiscs and effect on protein stability and activity

Rupinder Singh Jandu, Huaxu Yu, Zhiyu Zhao, Hai Tuong Le, Sehyeon Kim, Tao Huan, and Franck Duong van Hoa

Supplemental figures with corresponding titles and legends (non-Excel/CSV)

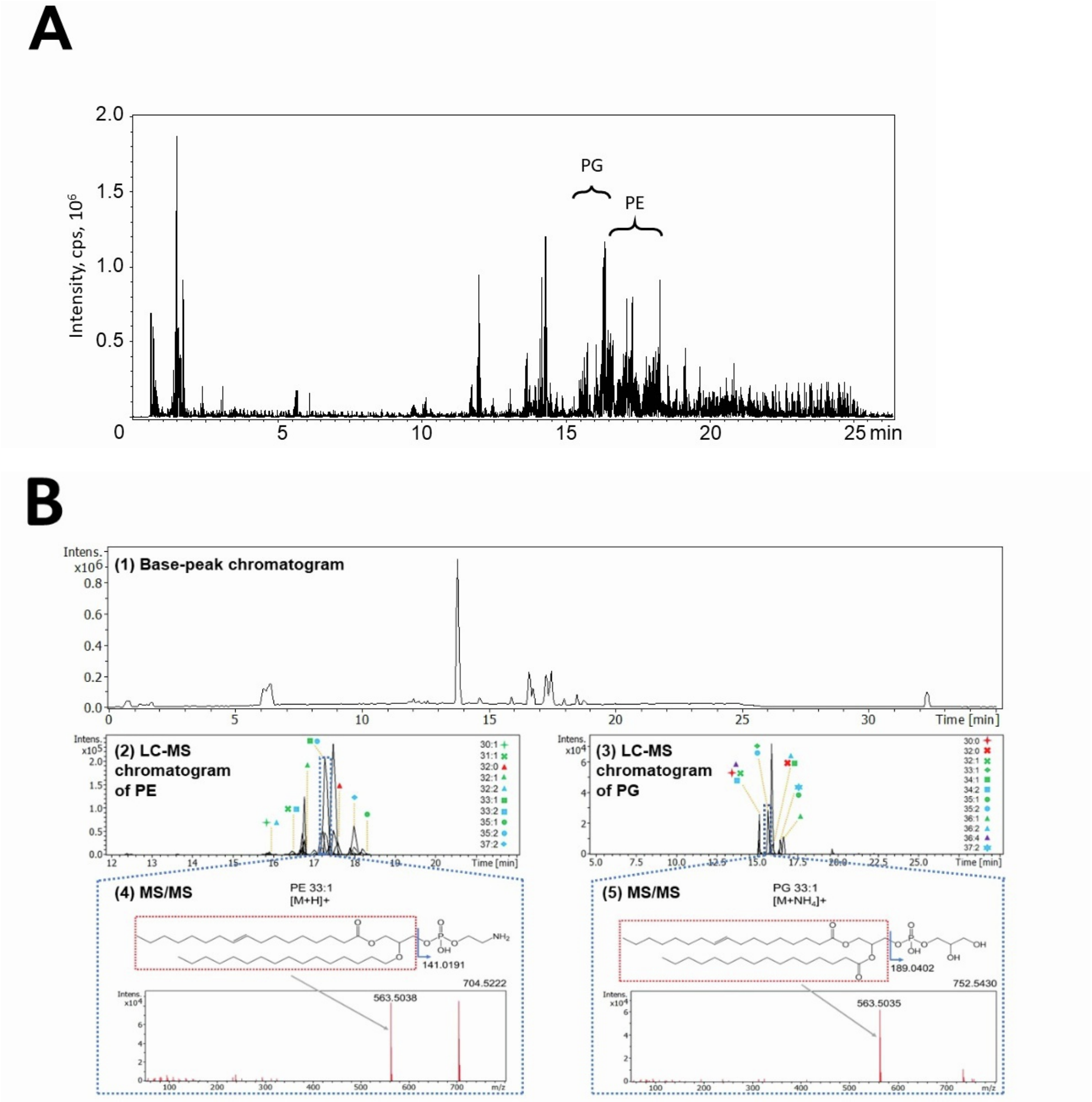

Supplementary Figure 1: LC-MS/MS analysis of lipids extracted from MsbA samples. Related to Figure 2.

(**A**) Representative total ion chromatogram of the DDC-MsbA preparation. **(B)** Example of lipid species identification and intensity quantification. (**1**) A base-peak chromatogram of extracted lipids. (**2**) and (**3**) Negative ion mass spectra across the elution time window corresponding to PE and PG lipid species. Icons highlight the variety of PE (**2**) and PG (**3**) phospholipids. (**4**) The molecular structures of PE 33:1 and PG 33:1, two abundant phospholipid species within the samples, along with their product ion (MS/MS) scan in negative ion mode. Fragmentation of the acyl chains from the phosphate head group can be visualized by the *m/z* ratios along with the measured intensity value.

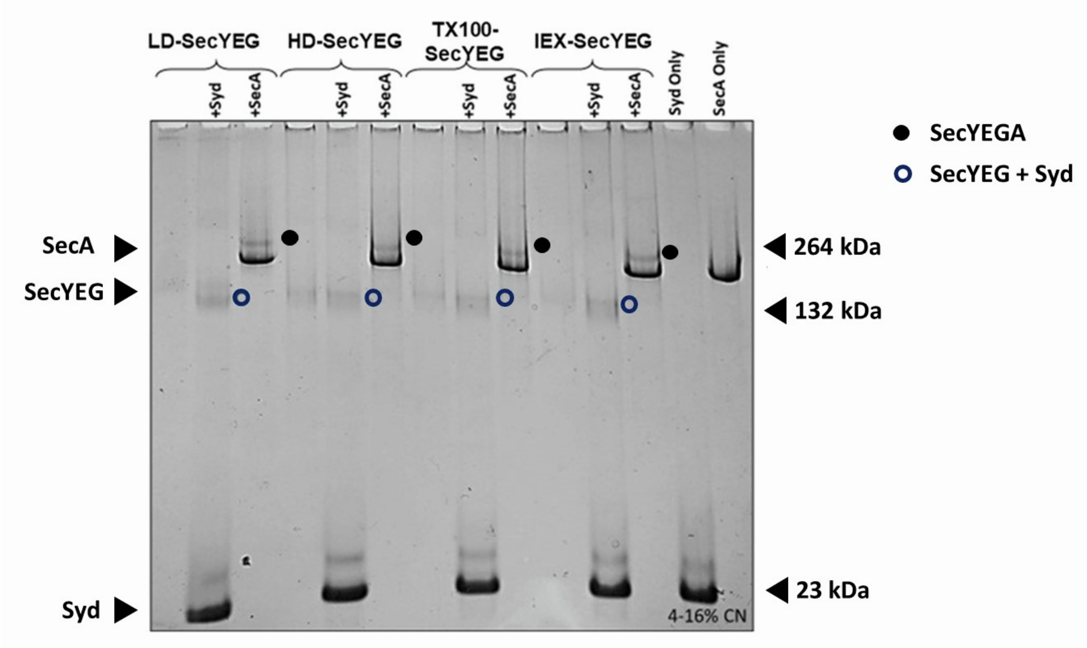

Supplementary Figure 2: Binding of SecA and Syd to the Peptidisc-SecYEG preparations. Related to Figure 7.

The Peptidisc-SecYEG preparations (0.5µg) were incubated with SecA or Syd (2 µg each) before analysis by 5-16% clear-native PAGE and Coomassie blue staining of the gel. The position of the SecYEG-SecA and SecYEG-Syd complex is indicated. The protein Syd is a SecY-binding protein. The binding of Syd ameliorates the electrophoretic mobility and detection of the SecYEG complex on this type of gel [59]. His-tagged Syd was purified as previously described [59].

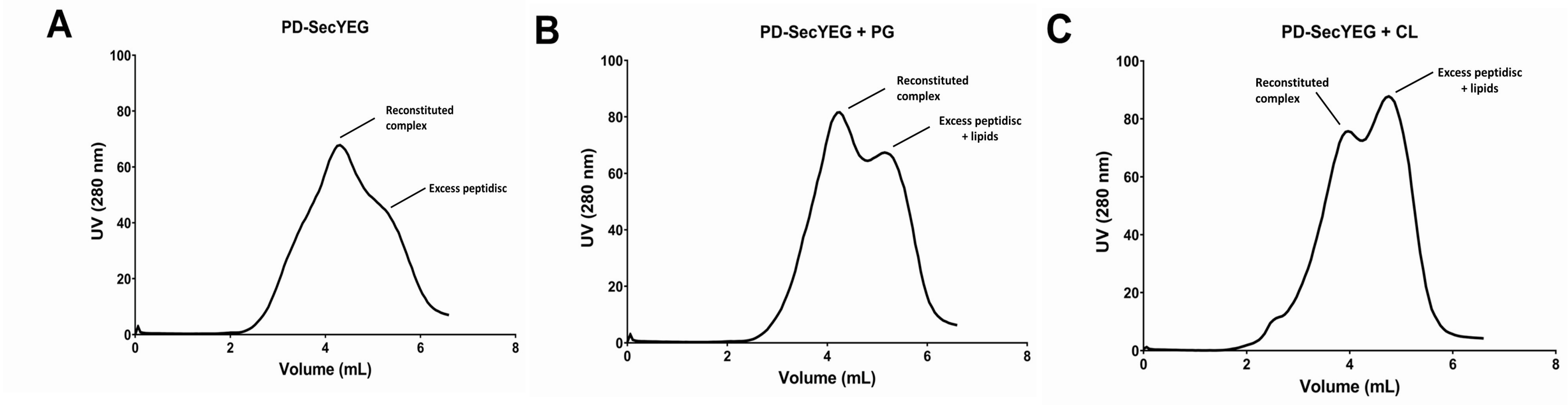

Supplementary Figure 3. Size-exclusion chromatography of the SecYEG complex in peptidisc with exogenous lipids. Related to Figure 8.

Size-exclusion chromatograms of the reconstitution of **(A)** SecYEG alone (PD-SecYEG), **(B)** SecYEG with PG (PD-SecYEG + PG), and **(C)** SecYEG with CL (PD-SecYEG + CL). The peak elution of the reconstituted SecYEG complex is indicated and corresponding fractions were pooled and analyzed as shown in Figure 8.
